# Relevance of VEGFA in rat livers subjected to partial hepatectomy under ischemia-reperfusion

**DOI:** 10.1007/s00109-019-01811-y

**Published:** 2019-06-29

**Authors:** Esther Bujaldon, María Eugenia Cornide-Petronio, José Gulfo, Floriana Rotondo, Cindy Ávalos de León, Elsa Negrete-Sánchez, Jordi Gracia-Sancho, Anna Novials, Mónica B. Jiménez-Castro, Carmen Peralta Uroz

**Affiliations:** 1 Institut d’Investigacions Biomèdiques August Pi i Sunyer (IDIBAPS), Barcelona, Spain; 2 Centro de Investigación Biomédica en Red de Enfermedades Hepáticas y Digestivas, Barcelona, Spain; 3 Liver Vascular Biology Research Group, IDIBAPS, CIBEREHD, Barcelona, Spain; 4 Diabetes and Obesity Research Laboratory, Institut d’Investigacions Biomèdiques August Pi i Sunyer (IDIBAPS), Barcelona, Spain; 5 Spanish Biomedical Research Center in Diabetes and Associated Metabolic Disorders (CIBERDEM), Barcelona, Spain; 6 Transplant Biomedicals S.L., Barcelona, Spain; 7 Facultad de Medicina, Universidad International de Cataluña, Barcelona, Spain

**Keywords:** Ischemia-reperfusion, Vascular endothelial growth factor A, Soluble vascular endothelial growth factor receptor 1, Liver, Steatosis, Adipose tissue

## Abstract

**Abstract:**

We examined the effects of VEGFA on damage and regeneration in steatotic and non-steatotic livers of rats submitted to PH under I/R, and characterized the underlying mechanisms involved. Our results indicated that VEGFA levels were decreased in both steatotic and non-steatotic livers after surgery. The administration of VEGFA increased VEGFA levels in non-steatotic livers, reducing the incidence of post-operative complications following surgery through the VEGFR2-Wnt2 pathway, independently of Id1. Unexpectedly, administration of VEGFA notably reduced VEGFA levels in steatotic livers, exacerbating damage and regenerative failure. After exogenous administration of VEGFA in steatotic animals, circulating VEGFA is sequestered by the high circulating levels of sFlt1 released from adipose tissue. Under such conditions, VEGFA cannot reach the steatotic liver to exert its effects. Consequently, the concomitant administration of VEGFA and an antibody against sFlt1 was required to avoid binding of sFlt1 to VEGFA. This was associated with high VEGFA levels in steatotic livers and protection against damage and regenerative failure, plus improvement in the survival rate via up-regulation of PI3K/Akt independently of the Id1-Wnt2 pathway. The current study highlights the different effects and signaling pathways of VEGFA in liver surgery requiring PH and I/R based in the presence of steatosis.

**Key messages:**

- VEGFA administration improves PH+I/R injury only in non-steatotic livers of Ln animals.
- VEGFA benefits are exerted through the VEGFR2-Wnt2 pathway in non-steatotic livers.
- In Ob rats, exogenous VEGFA is sequestered by circulating sFlt1, exacerbating liver damage.
- Therapeutic combination of VEGFA and anti-sFlt1 is required to protect steatotic livers.
- VEGFA+anti-sFlt1 treatment protects steatotic livers through a VEGFR2-PI3K/Akt pathway.

**Electronic supplementary material:**

The online version of this article (10.1007/s00109-019-01811-y) contains supplementary material, which is available to authorized users.

## Introduction

In clinical situations, partial hepatectomy (PH) under ischemia/reperfusion (I/R) is a common strategy to control bleeding during parenchymal dissection [1]. More than 20% of patients destined for liver resection present some degree of steatosis, a condition usually related to obesity [1–3], and the prevalence of steatosis is constantly increasing in society. Importantly, hepatic steatosis represents a major risk factor for liver surgery, being associated with high rates of complications and postoperative mortality after major liver resection [1, 4, 5].

A number of experimental studies on PH without I/R in steatotic and non-steatotic livers have shown that vascular endothelial growth factor A (VEGFA) levels are increased after surgery and that infusion of VEGFA can reduce injury and increase hepatocyte proliferation [6–9]. Furthermore, protective effects on damage have been reported regarding the role of VEGFA in non-steatotic livers in experimental models of I/R without PH [10, 11].

A number of studies have shown that VEGF receptor-2 (VEGFR2) is the principal mediator of several physiological and pathological effects of VEGFA [12–17]. It has also been shown that in non-steatotic livers undergoing PH without I/R, VEGFR2 activation is induced, initiating Id1 up-regulation and secretion of Wnt2 angiocrine factor [7]. Recent studies have suggested an important role of Wnt2 signaling in the proliferative response in non-steatotic livers undergoing I/R without PH [18].

As mentioned above, the role of VEGFA has been evaluated in PH without I/R and in I/R without PH, mainly focusing on non-steatotic livers. Nevertheless, the effect of VEGFA on liver regeneration and damage in conditions of PH under I/R has not been investigated. This scenario is addressed in the present study since PH under I/R is commonly used in the clinical practice to control bleeding during parenchymal dissection. We postulated that the expression and role of VEGFA as well as the mechanisms by which VEGFA might affect damage or regeneration might differ depending on the hepatic surgical conditions as well as the presence or absence of steatosis in the liver submitted to surgery. Consequently, strategies aimed at protecting the liver during surgery might be specific for each surgical procedure and for steatotic and non-steatotic livers.

Herein we examined VEGFA levels in rat steatotic and non-steatotic livers undergoing PH under I/R. We also investigated whether modulating the actions of VEGFA could protect both steatotic and non-steatotic livers against damage and regenerative failure following surgery. Finally, we investigated whether the VEGFR2-Id1-Wnt2 pathway is involved in the underlying action mechanisms of VEGFA in both steatotic and non-steatotic livers in an experimental model of PH under vascular occlusion, a liver surgery setting of potential clinical and scientific interest. In our opinion, the use of experimental surgical models that resemble as much as possible the clinical conditions in which the strategy is intended to be applied will lead to the translation of those strategies to clinical practice in the short term.

## Material and methods

### Experimental animals

Male homozygous obese (Ob) (400–450 g) and heterozygous lean (Ln) Zucker rats (350–400 g) and male Sprague Dawley (SD) and choline-deficient SD (CDD-SD) rats (350–380 g) were used. Ob Zucker and CDD-SD rats showed severe macrovesicular and microvesicular fatty infiltration in hepatocytes (60–70% steatosis) [19, 20].

### Experimental groups

#### Protocol 1. VEGFA impact and availability in Ln and Ob Zucker rats undergoing PH+I/R

1. Sham group (6 Ln and 6 Ob Zucker rats). Hepatic hilar vessels of animals were dissected.
2. PH+I/R group (6 Ln and 6Ob Zucker rats). Animals underwent partial hepatectomy (70%) under 60 min of ischemia [1, 20, 21].
3. PH+I/R+VEGFA group (6 Ln and 6 Ob Zucker rats). As in group 2, but treated with VEGFA (5 μg/kg i.v.) [6].
4. PH+I/R+VEGFA+anti-sFlt1 group (6 Ob Zucker rats). As in group 3, but treated with an antibody against soluble VEGF receptor-1 (sVEGFR1; also known as sFlt1) (0.6 mg/kg i.v.) [22].

#### Protocol 2. Role of adipose tissue in the circulating sFlt1 levels in Ln and Ob Zucker rats undergoing PH+I/R

5) Sham+LPT group (6 Ln and 6 Ob Zucker rats). Same as group 1, but mesenteric, perirenal, retroperitoneal, and epididymal adipose tissue were resected and extracted [1].
6) PH+I/R+LPT group (6 Ln and 6 Ob Zucker rats). Same as group 2, but mesenteric, perirenal, retroperitoneal, and epididymal adipose tissue were resected and extracted before starting PH+I/R [1].
7) PH+I/R+LPT+VEGFA group (6 Ln and 6 Ob Zucker rats). Same as group 3, but mesenteric, perirenal, retroperitoneal, and epididymal adipose tissue were resected and extracted before starting PH+I/R [1].

#### Protocol 3. Underlying mechanisms of VEGFA in Ln and Ob Zucker rats undergoing PH+I/R

8) PH+I/R+VEGFA+anti-VEGFR2 group (6 Ln Zucker rats). As in group 3, but treated with ZD6474, a potent inhibitor of VEGFR2 (2.5 mg/kg i.v.) [23].
9) PH+I/R+Wnt2 group (6 Ln Zucker rats). As in group 2, but treated with Wnt2 (5 ng/kg, i.v.) [24].
10) PH+I/R+VEGFA+anti-sFlt1+anti-VEGFR2 group (6 Zucker Ob rats). As in group 4, but treated with ZD6474, a potent inhibitor of VEGFR2 (2.5 mg/kg i.v.) [23].
11) PH+I/R+VEGFA+anti-sFlt1+PI3K/Akt-inh group (6 Ob Zucker rats). As in group 4, but treated with LY294002, a potent inhibitor of Akt (0.5 mg/kg i.p.), and Wortmannin, a potent inhibitor of PI3K (1 mg/kg i.p.) [25].

#### Protocol 4. VEGFA impact and availability in SD and CDD-SD rats undergoing PH+I/R

12) Sham group (6 SD and 6 CDD-SD rats). Hepatic hilar vessels of animals were dissected.
13) PH+I/R group (6 SD and 6 CDD-SD rats). Animals underwent partial hepatectomy (70%) under 60 min of ischemia following the same surgical procedure as in group 2 of protocol 1 [1, 20, 21].
14) PH+I/R+VEGFA group (6 SD and 6 CDD-SD rats). As in group 13, but treated with VEGFA (5 μg/kg i.v.) [6].
15) PH+I/R+VEGFA+anti-sFlt1 group (6 CDD-SD rats). As in group 14, but treated with an antibody against soluble VEGF receptor-1 (sVEGFR1; also known as sFlt1) (0.6 mg/kg i.v.) [22].

The sample collection and the measurements for protocols 1–4 at the corresponding reperfusion times are shown in Online Resource 1.

## Results

### VEGFA impact in Ln and Ob Zucker rats undergoing PH+I/R

**Fig. 1:**
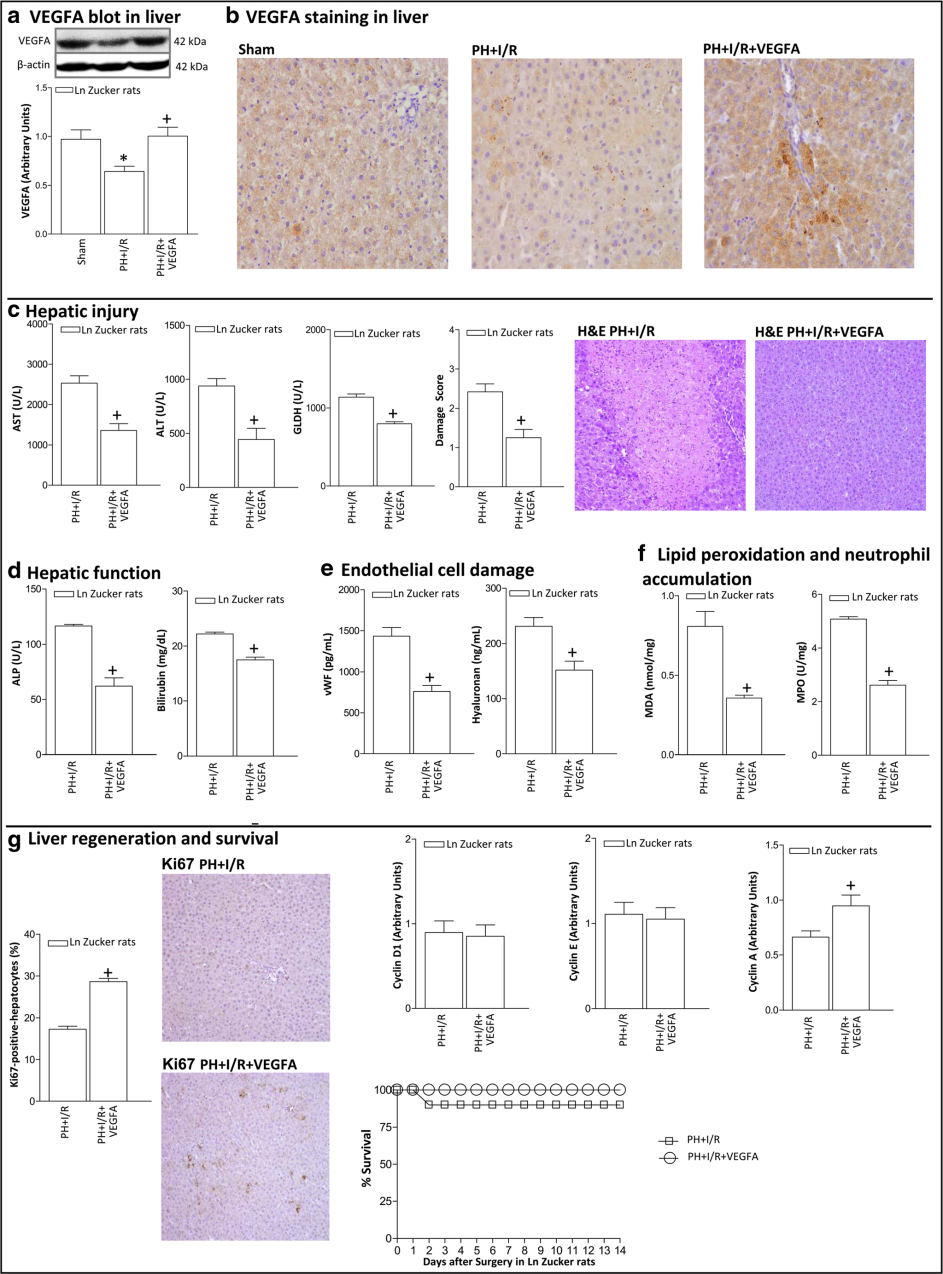
VEGFA protein levels and effects of VEGFA administration on hepatic damage and function, liver regeneration 24 h after surgery, and survival rate in Ln Zucker rats. **A** Protein levels of VEGFA in liver. Representative Western blots at the top and densitometric analysis at the bottom. **B** Representative photomicrographs of VEGFA immunohistochemical positivity in liver (× 20). **C** Hepatic injury (plasma AST, ALT, and GLDH levels; damage score and representative photomicrographs of necrosis (H&E stain, × 10)). **D** Hepatic function (ALP and bilirubin levels). **E** Endothelial cell damage (vWF and HA levels). **F** Lipid peroxidation (MDA levels) and neutrophil accumulation (MPO levels). **G** Hepatic regeneration (percentage of Ki67-positive-hepatocytes and representative photomicrographs of Ki67 immunohistochemical positivity (× 10); cyclin D1, E, and A levels). Survival of Ln Zucker animals was monitored (postoperative day 14) (**G**). *P < 0.05 versus sham; +*P* < 0.05 versus PH+I/R

In Ln Zucker animals, hepatic VEGFA protein levels were decreased in the PH+I/R group compared to those of the Sham group, as demonstrated by VEGFA immunoblot (Fig. 1A) and immunohistochemistry (Fig. 1B). Administration of VEGFA in Ln animals (PH+I/R+VEGFA group) increased hepatic VEGFA levels compared with the PH+I/R group (Fig. 1A). Accordingly, PH+I/R+VEGFA in non-steatotic livers exhibited higher VEGFA immunohistochemical staining in both hepatocytes and in non-parenchymal cells than that found in the PH+I/R group (Fig. 1B). VEGFA administration (PH+I/R+VEGFA group) protected against hepatic damage as indicated by the reduced transaminases, GLDH, damage score values, and the extent and number of necrotic areas in non-steatotic livers when compared with the PH+I/R group (Fig. 1C). Liver function (measured as ALP and bilirubin levels) and endothelial cell damage (measured by vWF and HA levels) were reduced by VEGFA treatment (Figs. 1D–E). This was associated with a reduction in oxidative stress and neutrophil accumulation (Fig. 1F).

In terms of liver regeneration, the administration of VEGFA (PH+I/R+VEGFA group) increased the percentage of Ki67-positive-hepatocytes in non-steatotic livers (Fig. 1G). The effect of VEGFA on the progression of the cellular cycle was also examined. The levels of cyclin D1, which is necessary for G1 phase progression [26], and cyclin E, which is induced during G1 and mediates transition into the S phase [27] were unchanged in non-steatotic livers of the PH+I/R+VEGFA group with respect to those of the PH+I/R group (Fig. 1G). However, hepatic cyclin A expression, which is necessary for S phase progression [28], was higher in non-steatotic livers of the PH+I/R+VEGFA group when compared with the PH+I/R group (Fig. 1G). The administration of VEGFA (PH+I/R+VEGFA group) reduced lethality in Ln animals when compared with the PH+I/R group (Fig. 1G).

**Fig. 2:**
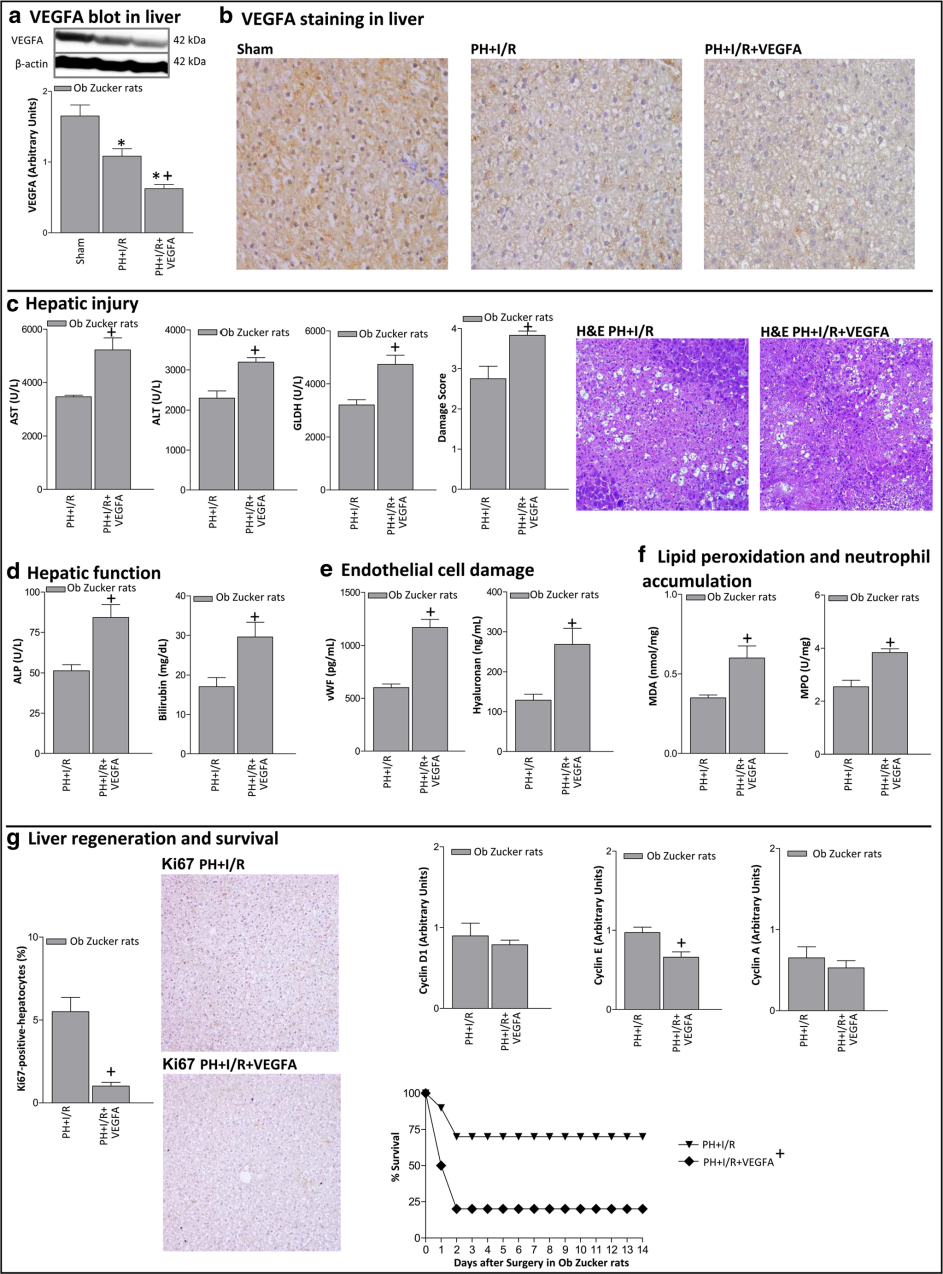
VEGFA protein levels and effects of VEGFA administration on hepatic damage and function, liver regeneration 24 h after surgery, and survival rate in Ob Zucker rats. **A** Protein levels of VEGFA in liver. Representative Western blots at the top and densitometric analysis at the bottom. **B** Representative photomicrographs of VEGFA immunohistochemical positivity in liver (× 20). **C** Hepatic injury (plasma AST, ALT, and GLDH levels; damage score and representative photomicrographs of necrosis (× 10). **D** Hepatic function (ALP and bilirubin levels). **E** Endothelial cell damage (vWF and HA levels). **F** Lipid peroxidation (MDA levels) and neutrophil accumulation (MPO levels). **G** Hepatic regeneration (percentage of Ki67-positive-hepatocytes and representative photomicrographs of Ki67 immunohistochemical positivity (× 10); cyclin D1, E, and A levels). Survival of Ob Zucker animals was monitored (postoperative day 14) (**G**). *P < 0.05 versus sham; +*P* < 0.05 versus PH+I/R

In Ob Zucker animals, reductions in hepatic VEGFA levels were observed in steatotic livers of the PH+I/R when compared with the Sham group (Figs. 2A–B). Unexpectedly, administration of VEGFA in Ob Zucker animals (PH+I/R+VEGFA group) further reduced hepatic VEGFA levels in steatotic livers compared with the PH+I/R group (Figs. 2A–B). With regard to hepatic damage, the administration of VEGFA in steatotic livers (PH+I/R+VEGFA group) increased transaminases, GLDH, ALP, and bilirubin (Figs. 2C–D). This was associated with high levels of both vWF and HA (Fig. 2E), exacerbated oxidative stress, and neutrophil accumulation (Fig. 2F). This increased the damage score, extending the necrotic areas ordinarily seen after PH+I/R (Fig. 2C). The number of Ki67-positive hepatocytes and cyclin E levels in steatotic livers of the PH+I/R+VEGFA group were lower than in the PH+I/R group (Fig. 2G). These deleterious effects of VEGFA on hepatic damage and regenerative failure in Ob Zucker rats were also observed 72 h after surgery (Online Resource 2). However, the administration of VEGFA (PH+I/R+VEGFA group) reduced the survival rate in Ob Zucker animals at 14 days when compared with the results of the PH+I/R group (Fig. 2G).

### Relevance of VEGFA availability in Ln and Ob Zucker rats undergoing PH+I/R

**Fig. 3:**
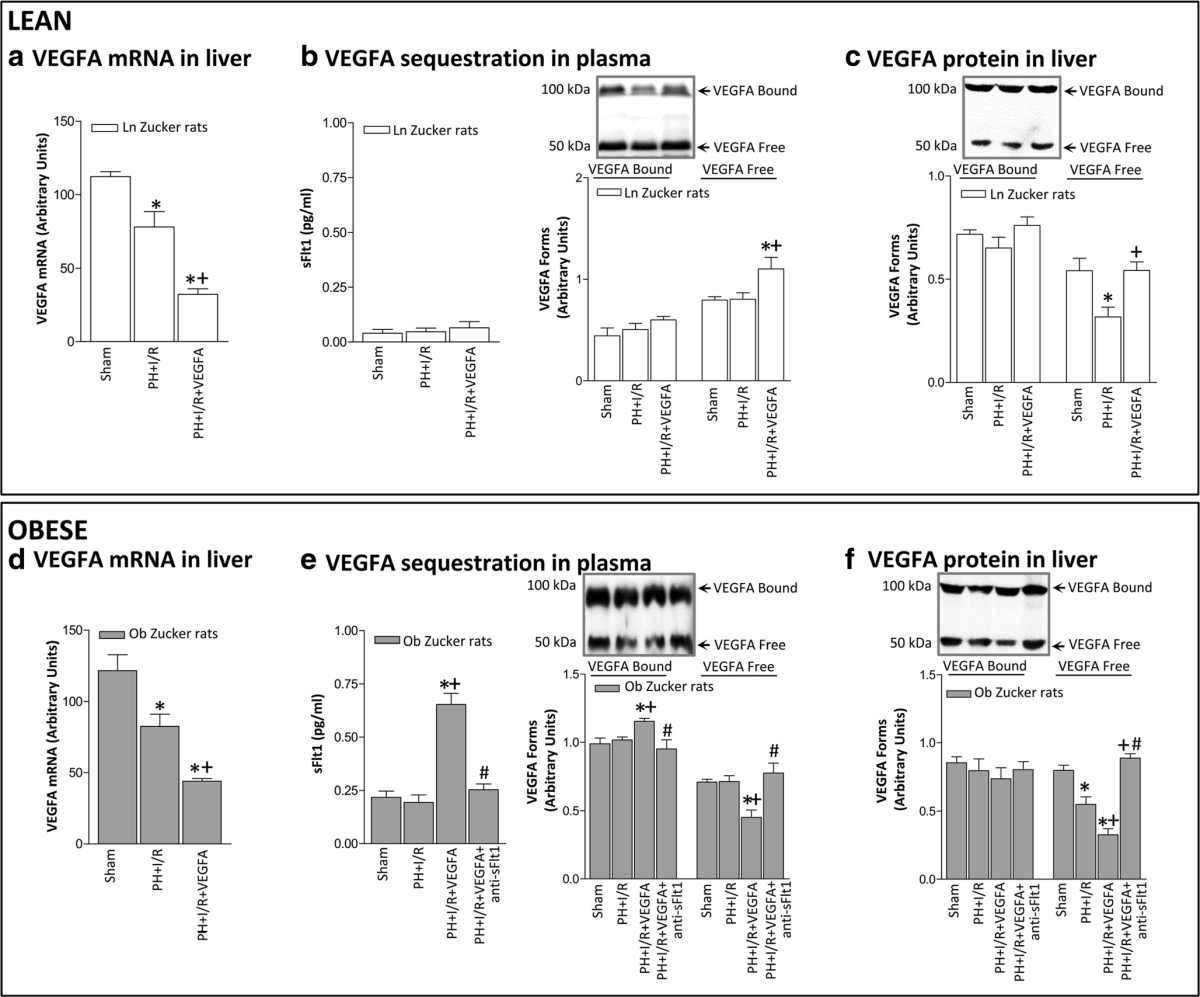
VEGFA availability in Ln and Ob Zucker rats 24 h after surgery. **A** Hepatic mRNA VEGFA in non-steatotic livers. **B** Plasma levels of sFlt1 and the bound and free forms of VEGFA in Ln Zucker animals. **C** Hepatic protein levels of VEGFA in non-steatotic livers. **D** Hepatic mRNA VEGFA in steatotic livers. **E** Plasma levels of sFlt1 and the bound and free forms of VEGFA in Ob Zucker animals. **F** Hepatic protein levels of VEGFA in steatotic livers. *P < 0.05 versus sham; +*P* < 0.05 versus PH+I/R; #*P* < 0.05 versus PH+I/R+VEGFA

**Fig. 4:**
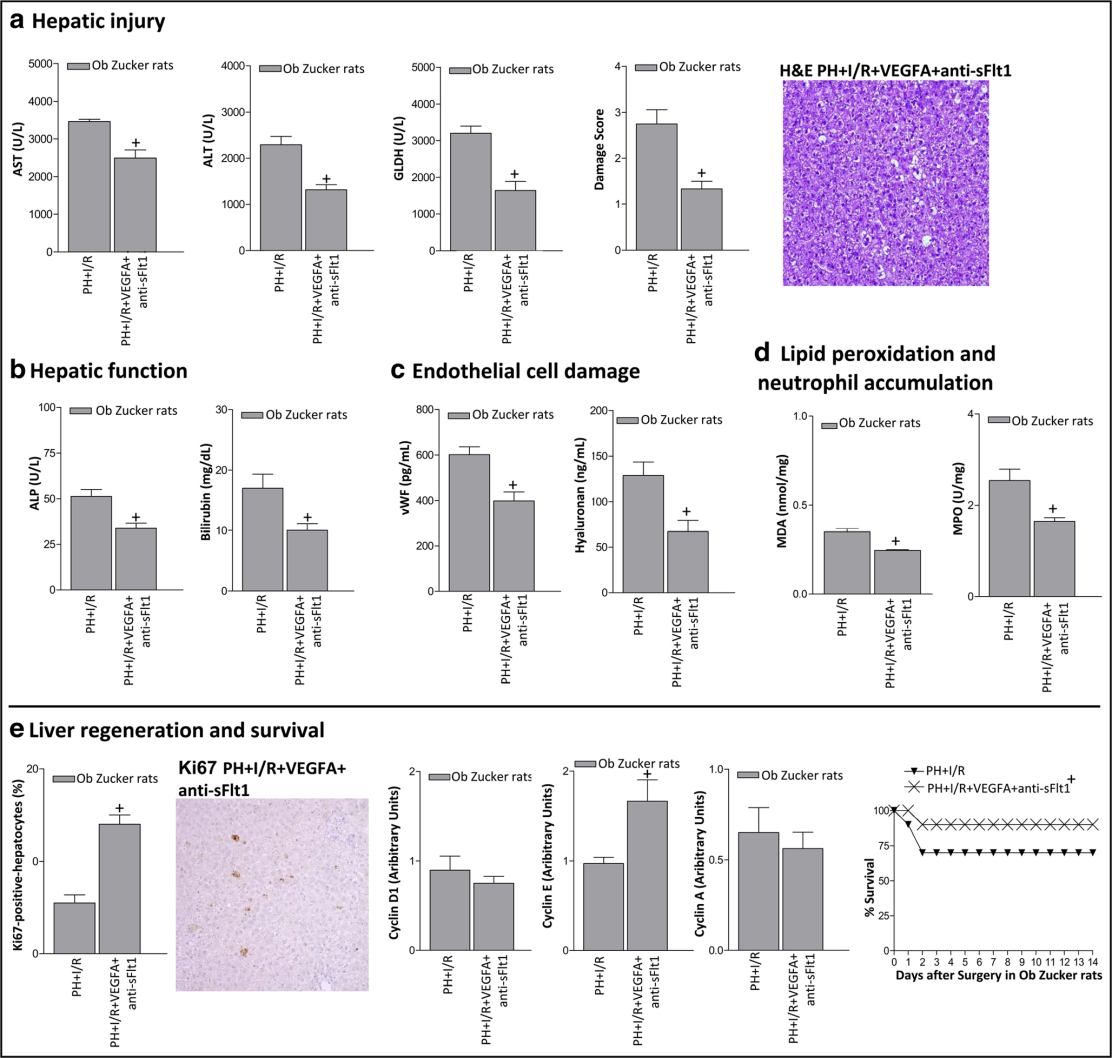
Effects of the concomitant administration of VEGFA and antibodies against anti-sFlt1 on hepatic damage and regeneration 24 h after surgery and survival rate in Ob Zucker rats. **A** Hepatic injury (plasma AST, ALT, and GLDH levels; damage score and representative photomicrograph of necrosis (× 10)). **B** Hepatic function (ALP and bilirubin levels). **C** Endothelial function (vWF and HA levels). **D** Lipid peroxidation (MDA levels) and neutrophil accumulation (MPO levels). **E** Hepatic regeneration (percentage of Ki67 positive-hepatocytes and representative photomicrograph of Ki67 immunohistochemical positivity (× 10); cyclin D1, E, and A levels). Survival of Ob Zucker animals was monitored (postoperative day 14) (**E**). +*P* < 0.05 versus PH+I/R

We next try to explain why, in contrast to non-steatotic livers, the administration of VEGFA reduced VEGFA levels in steatotic livers when compared with the PH+I/R group (Fig. 3). One hypothesis involved a reduction in VEGFA synthesis in steatotic livers compared with non-steatotic livers. However, this did not seem to be the case. Indeed, our results indicated that VEGFA administration (PH+I/R+VEGFA group) reduced mRNA levels of VEGFA in both non-steatotic and steatotic livers when compared with the PH+I/R group, being its mRNA expression comparable in non-steatotic (Fig. 3A) and steatotic (Fig. 3D) livers (*P* > 0.05, not significant). Since sFlt1 is capable of sequestering and thus determining the circulating levels of VEGFA, and thereby preventing its signal transduction [29–31], and considering that plasma sFlt1 levels are elevated in different liver diseases [32, 33], an attempt was made to discern whether the differences in VEGFA levels in non-steatotic and steatotic livers resulting from the exogenous administration of VEGFA may be explained, at least partially by potential differences in the circulating levels of sFlt1. We hypothesized that after exogenous administration of VEGFA in Ob Zucker animals, circulating VEGFA is sequestered by sFlt1 and consequently VEGFA cannot reach the liver to exert its effects. Firstly, we measured plasma levels of sFlt1 and VEGFA corresponding to bound forms (100–130 kDa) and free forms (45–50 kDa). Of note, plasma sFlt1 levels in the PH+I/R group were lower in Ln than in Ob Zucker animals (Fig. 3B, E). The administration of VEGFA in Ln Zucker animals (PH+I/R+VEGFA group) did not induce changes in either circulating sFlt1 or circulating VEGFA corresponding to the bound form when compared with the PH+I/R group (Fig. 3B). However, the level of circulating free VEGFA was higher in Ln animals of the PH+I/R+VEGFA group when compared with the PH+I/R group (Fig. 3B). This was associated with high VEGFA-free form levels in non-steatotic livers (Fig. 3C). In Ob Zucker animals, the administration of VEGFA (PH+I/R+VEGFA group) increased circulating sFlt1 when compared with the PH+I/R group (Fig. 3E). Under these conditions, the circulating VEGFA bound was higher whereas the circulating VEGFA free form was lower than in the PH+I/R group (Fig. 3E). This was associated with reduced VEGFA levels in steatotic livers (Fig. 3F). To reinforce our hypothesis based on the VEGFA-sFlt1 complex, further experiments were carried out. We co-administered VEGFA with an antibody against sFlt1 (PH+I/R+VEGFA+anti-sFlt1 group) to avoid binding of sFlt1 to VEGFA. Under these conditions, VEGFA should reach the liver to exert its effects. Interestingly, our results indicated that Ob Zucker animals in the PH+I/R+VEGFA+anti-sFlt1 group exhibited reduced plasma levels of VEGFA bound form and increased plasma levels of the free form when compared with the results of the PH+I/R+VEGFA group (Fig. 3E). Of interest, the PH+I/R+VEGFA+anti-sFlt1 group exhibited higher levels of VEGFA free form in steatotic livers than those of the PH+I/R+VEGFA group (Fig. 3F). Importantly, under these conditions, VEGFA did confer protection in steatotic livers. Indeed, the PH+I/R+VEGFA+anti-sFlt1 group showed reduced hepatic injury and improved liver functionality and survival rate when compared with the PH+I/R group (Fig. 4A–E). Likewise, Ki67-positive hepatocytes showed significant increase, associated with high cyclin E levels (Fig. 4E). Thus, the blockade of sFlt1 action in Ob Zucker animals treated with VEGFA (PH+I/R+VEGFA+anti-sFlt1 group) allowed VEGFA to reach the liver and consequently to protect steatotic livers against damage and regenerative failure.

### Role of adipose tissue in the circulating sFlt1 levels in Ln and Ob Zucker rats undergoing PH+I/R

**Fig. 5:**
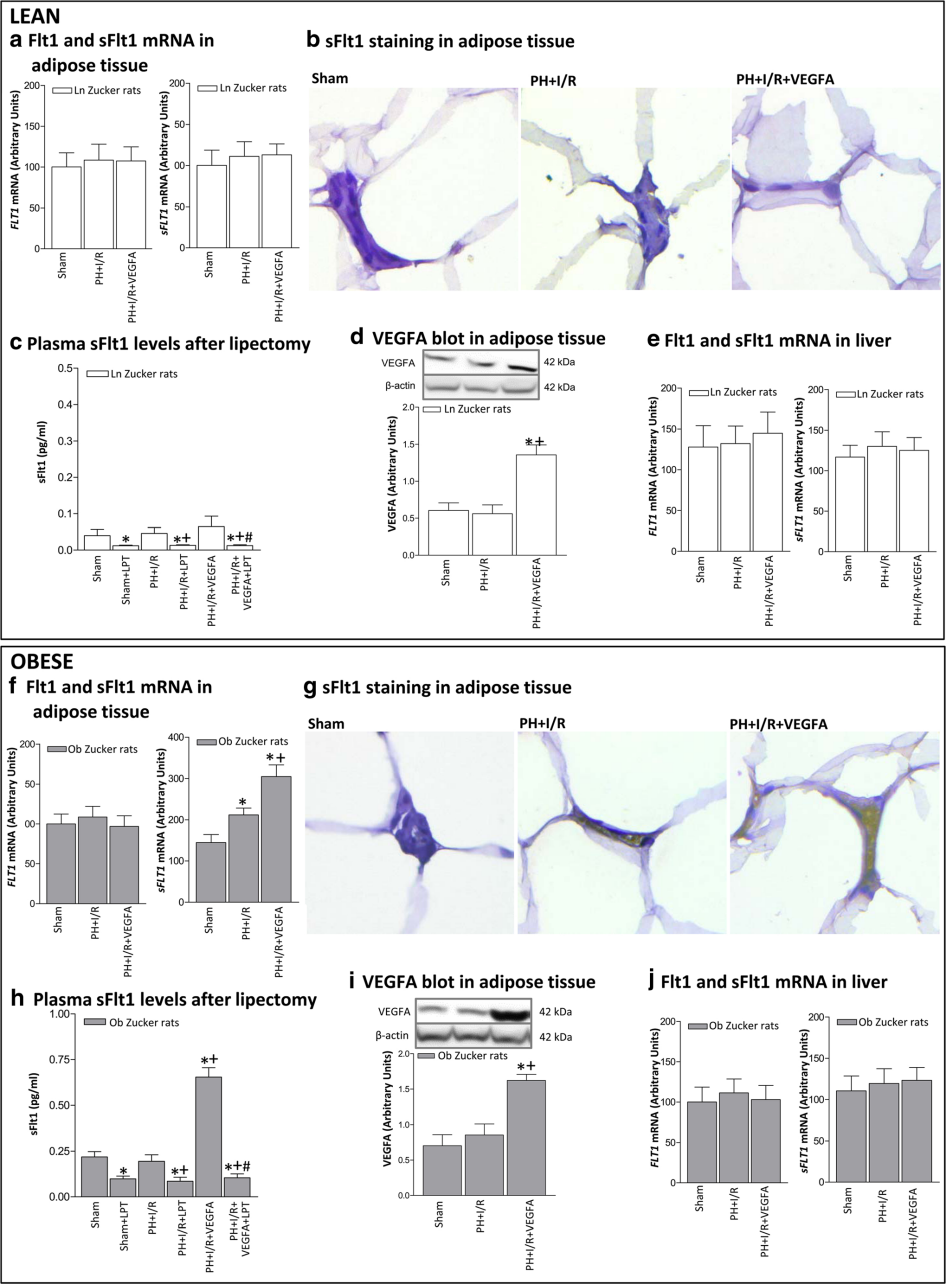
Role of adipose tissue in the circulating sFlt1 levels in Ln and Ob Zucker rats 24 h after surgery. **A** mRNA expression levels of Flt1 and sFlt in adipose tissue of Ln Zucker animals. **B** Representative photomicrographs of Flt1 immunohistochemical negativity in adipocyte membranes and sFlt1 immunohistochemical negativity in stromal space of adipose tissue of Ln Zucker animals (× 40). **C** Plasma sFlt1 levels in Ln Zucker animals subjected to lipectomy. **D** Protein levels of VEGFA in adipose tissue of Ln Zucker animals. Representative Western blots at the top and densitometric analysis at the bottom. **E** mRNA expression levels of Flt1 and sFlt in liver of Ln Zucker animals. **F** mRNA expression levels of Flt1 and sFlt in adipose tissue of Ob animals. **G** Representative photomicrographs of Flt1 immunohistochemical negativity in adipocyte membranes in all groups and sFlt1 immunohistochemical positivity in stromal space of adipose tissue only in PH+I/R and PH+I/R+VEGFA groups of Ob Zucker animals (× 40). **H** Plasma sFlt1 levels in Ob Zucker animals subjected to lipectomy. **I** Protein levels of VEGFA in adipose tissue of Ob Zucker animals. Representative Western blots at the top and densitometric analysis at the bottom. **J** mRNA expression levels of Flt1 and sFlt in liver of Ob Zucker animals. *P < 0.05 versus sham; +*P* < 0.05 versus PH+I/R; #*P* < 0.05 versus PH+I/R+VEGFA

Given our results indicating that plasma sFlt1 levels were lower in Ln than in Ob Zucker rats and in view of previous reports indicating that obesity impairs adipocyte function and secretion of mediators derived from adipose tissue to circulation [34–36], we next evaluated the potential contribution of adipose tissue in the circulating sFlt1 levels in Ln and Ob Zucker rats undergoing PH+I/R (Fig. 5). Thus, in Ln animals, Flt1 and sFlt1 levels in adipose tissue of the PH+I/R group were similar to those of the Sham group, as demonstrated by mRNA expression (Fig. 5A) and immunohistochemistry analysis (Fig. 5B). In Ob animals, the PH+I/R group exhibited sFlt1 immunohistochemical positivity in stromal space of adipose tissue and higher mRNA levels of sFlt1 in adipose tissue than those of the Sham group, whereas Flt1 levels were unchanged (Fig. 5F, G). Next, Ln and Ob animals were submitted to interventions based on the elimination of peripheral adipose store, and the levels of circulating sFlt1 were evaluated. Removal of the peripheral adipose tissue in Ln and Ob animals of the Sham and PH+I/R group (Sham+LPT and PH+I/R+LPT) lowered plasma sFlt1 levels (Fig. 5C, H). As in the case of circulating sFlt1, the sFlt1 levels in adipose tissue of the Sham and PH+I/R groups were lower in Ln than in Ob animals (Fig. 5A, F). In Ln animals, VEGFA administration (PH+I/R+VEGFA group) reached the adipose tissue since VEGFA levels rose compared with the PH+I/R group (Fig. 5D) but did not induce changes in either adipose tissue or circulating sFlt1 levels (Figs. 5A–C). This scenario was different in the case of Ob animals, since VEGFA administration (PH+I/R+VEGFA group) reached the adipose tissue (Fig. 5I) and increased sFlt1 as shown by the increases in both the mRNA expression of sFlt1 levels (Fig. 5F) and sFlt1 immunohistochemical staining in the stromal space when compared with the PH+I/R group (Fig. 5G). Under these conditions, circulating sFlt1 levels were also increased (Fig. 5H). However, this increase in circulating sFlt1 levels was no longer observed when adipose tissue was removed (PH+I/R+VEGFA+LPT group). Our results confirmed the minor role of the liver in the circulating sFlt1 levels in Ln and Ob animals undergoing PH+I/R since hepatic Flt1 and sFlt1 levels were unchanged in the groups in the study (Sham, PH+I/R and PH+I/R+LPT groups) (Fig. 5E, J).

### Underlying mechanisms of VEGFA in Ln and Ob Zucker rats undergoing PH+I/R

**Fig. 6:**
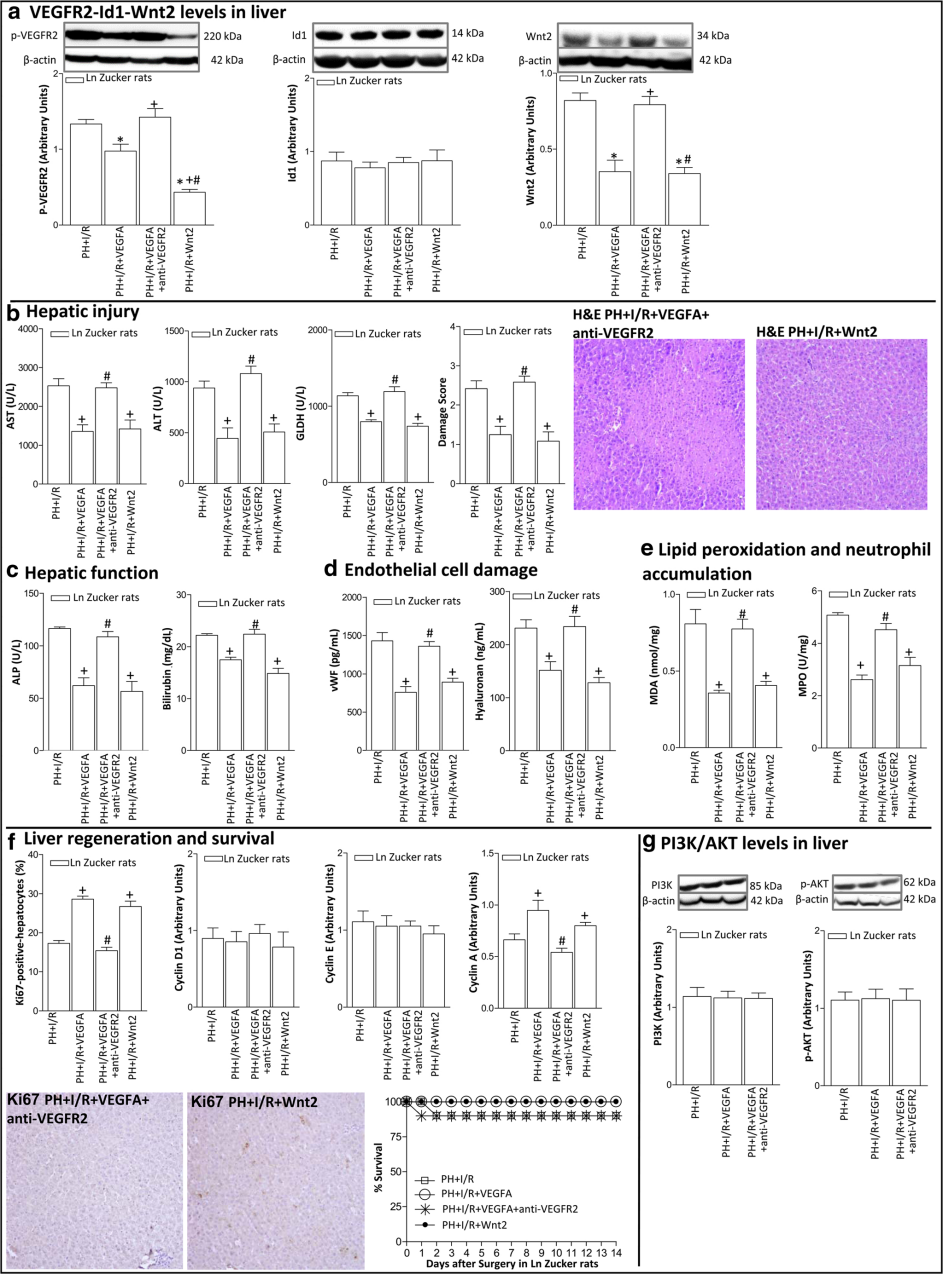
Underlying action mechanisms of VEGFA in non-steatotic livers of Ln Zucker rats 24 h after surgery. **A** Protein levels of VEGFR2, Id1, and Wnt2. Representative Western blots at the top and densitometric analysis at the bottom. **B** Hepatic injury (plasma AST, ALT, and GLDH levels; damage score and representative photomicrographs of necrosis (× 10)). **C** Hepatic function (ALP and bilirubin levels). **D** Endothelial cell damage (vWF and HA levels). **E** Lipid peroxidation (MDA levels) and neutrophil accumulation (MPO levels). **F** Hepatic regeneration (percentage of Ki67 positive-hepatocytes and representative photomicrographs of Ki67 immunohistochemical positivity (× 10); cyclin D1, E, and A levels). **G** Protein levels of PI3K and p-AKT. Representative Western blots at the top and densitometric analysis at the bottom. Survival of Ln Zucker animals, per group was monitored (postoperative day 14) (Fig. 5F). *P < 0.05 versus sham; +*P* < 0.05 versus PH+I/R; #*P* < 0.05 versus PH+I/R+VEGFA

Next, we investigated whether the effects of VEGFA on hepatic damage and regenerative response were mediated though the VEGFR2-Id1-Wnt2 pathway. In non-steatotic livers, VEGFR2 levels in the PH+I/R group were lower than those in the Sham group (Fig. 6A). Id1 levels remained unaltered in all groups, whereas this was not the case for Wnt2. Indeed, Wnt2 levels in the PH+I/R group were lower than those in the Sham group (Fig. 6A). The protection against damage and regenerative failure in non-steatotic livers resulting from the administration of VEGFA (PH+I/R+VEGFA group) was associated with increased expression of VEGFR2 and Wnt2 (Fig. 6A). Inhibition of VEGFA action using an antibody against VEGFR2 (PH+VEGFA+anti-VEGFR2 group) abolished the benefits derived from VEGFA in non-steatotic livers resulting in similar Wnt2 levels (Fig. 6A) and parameters of hepatic damage (Figs. 6B–E), regeneration, and survival rate (Fig. 6F) to those of the PH+I/R group. The relevance of Wnt2 to hepatic damage and regenerative response was additionally confirmed demonstrating that recombinant Wnt2 administration (PH+I/R+Wnt2 group) reduced hepatic injury and improved liver regeneration in non-steatotic livers when compared with the PH+I/R group (Fig. 6B–F). The PI3K/Akt pathway has been implicated in the effects of VEGFA in isolated hepatic cells [12]. However, this does not seem to be the case in non-steatotic livers undergoing surgery, since VEGFA administration (PH+I/R+VEGFA and PH+I/R+VEGFA+anti-VEGFR2 groups) was not associated with changes in the protein expression of PI3K/Akt when compared with the PH+I/R group (Fig. 6G).

**Fig. 7:**
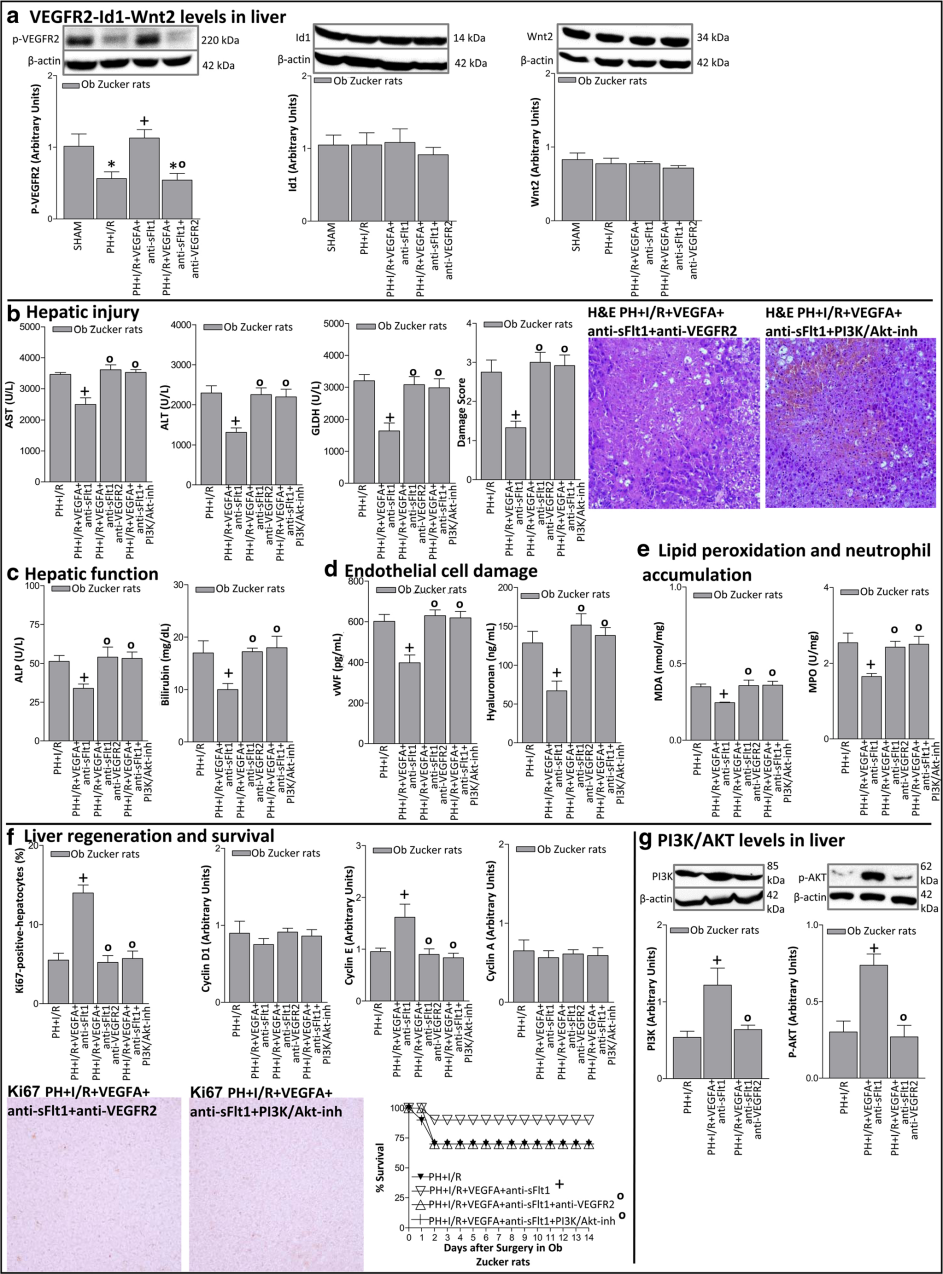
Underlying action mechanisms of VEGFA in steatotic livers of Ob Zucker rats 24 h after surgery. **A** Protein levels of VEGFR2, Id1, and Wnt2. Representative Western blots at the top and densitometric analysis at the bottom. **B** Hepatic injury (plasma AST, ALT, and GLDH levels; damage score and representative photomicrograph of necrosis (× 10)). **C** Hepatic function (ALP and bilirubin levels). **D** Endothelial cell damage (vWF and HA levels). **E** Lipid peroxidation (MDA levels) and neutrophil accumulation (MPO levels). **F** Hepatic regeneration (percentage of Ki67 positive-hepatocytes and representative photomicrograph of Ki67 immunohistochemical positivity (× 10); cyclin D1, E, and A levels). **G** Protein levels of PI3K and p-AKT. Representative Western blots at the top and densitometric analysis at the bottom. Survival of Ob animals, per group was monitored (postoperative day 14) (Fig. 6F). *P < 0.05 versus sham; +*P* < 0.05 versus PH+I/R; °*P* < 0.05 versus PH+I/R+VEGFA+anti-sFlt1

In steatotic livers, VEGFR2 levels in the PH+I/R+VEGFA+anti-sFlt1 group were increased with respect to those of the PH+I/R group (Fig. 7A). In addition, inhibition of VEGFA action in the PH+I/R+VEGFA+anti-sFlt1 group using antibodies against VEGFR2 (PH+I/R+VEGFA+anti-sFlt1+anti-VEGFR2 group) abolished the protection against hepatic injury and regeneration resulting from the administration of VEGFA+sFlt1 in steatotic livers (Fig. 7B–F). There was no change in either Id1 or Wnt2 when compared with the results of the PH+I/R or PH+I/R+VEGFA+sFlt1 groups (Fig. 7A). All these results indicate the minor role of the Id1-Wnt2 signaling pathways in the action of VEGFA+anti-sFlt1 in steatotic livers. Importantly, increased PI3K/Akt expression was observed in steatotic livers of the PH+I/R+VEGFA+anti-sFlt1 group when compared with the results of the PH+I/R group (Fig. 7G). The increase in PI3K/Akt expression was abolished when VEGFA was inhibited using an antibody against VEGFR2 (PH+I/R+VEGFA+anti-sFlt1+anti-VEGFR2 group) (Fig. 7G). Moreover, the benefits of VEGFA in the PH+I/R+VEGFA+anti-sFlt1 group were no longer observed when the PI3K/Akt signaling pathway was inhibited (PH+I/R+VEGFA+anti-sFlt1+PI3K/Akt-inh group) (Fig. 7B–F).

### The impact of VEGFA in SD and CDD-SD rats undergoing PH+I/R

As in the genetic obesity model (Zucker rats), in CDD-SD rats, hepatic VEGFA protein levels were lower in the PH+I/R group than in the Sham group (Online Resource 3). In addition, our results revealed an improvement in damage and regenerative process of PH+I/R+VEGFA in non-steatotic livers from SD rats when compared with the PH+I/R group. On the other hand, in steatotic livers from CDD-SD rats, VEGFA administration increased sFlt1 levels in adipose tissue and circulation, and exacerbated hepatic damage and regenerative failure. In CDD-SD rats, the co-administration of VEGFA+anti-sFlt1 was required to confer protection against damage and regenerative failure. The underlying protective mechanisms of VEGFA in an experimental model of steatosis induced by CDD were similar to those described in the genetic obesity model (data not shown).

## Discussion

To the best of our knowledge, no previous studies have reported changes in VEGFA levels in both steatotic and non-steatotic livers submitted to PH under I/R, a procedure commonly applied in surgery to reduce bleeding. Herein, we observed reduced mRNA and protein levels of VEGFA in steatotic and non-steatotic livers. In our study, VEGFA administration exhibited differential effects on damage and regeneration in steatotic and non-steatotic livers. Thus, under PH+I/R conditions, VEGFA administration in Ln Zucker animals increased VEGFA in non-steatotic livers and decreased hepatic injury and regeneration failure. On the other hand, VEGFA administration in Ob Zucker animals undergoing surgery reduced VEGFA levels in steatotic livers and negatively affected damage and regeneration. These effects of VEGFA have also been demonstrated in an experimental model of steatosis induced by CDD.

An attempt was made to discern the role of adipose tissue in the circulating sFlt1 levels in Ln and Ob Zucker rats undergoing PH+I/R, for the following reasons: functional differences between lean and obese adipose tissue have been extensively reported [37–39], obesity is associated with oxidative stress and inflammatory response in adipose tissue [40–42], and impairs adipocyte function and secretion of mediators derived from adipose tissue to circulation [34–36]. Our results in Zucker rats indicate the following: (a) adipose sFlt1 levels were higher in Ob than in Ln animals, (b) the increase in adipose tissue sFlt1 levels as consequence of PH+I/R occurred only in Ob animals, and (c) the adipose tissue was involved as a source of circulating sFlt1 levels. VEGFA treatment increased sFlt1 in adipose tissue only in Ob animals, and this resulted in the release of sFlt1 from adipose tissue to the circulation. Thus, we believe that the high levels of circulating sFlt1 (derived from adipose tissue) in Ob Zucker animals undergoing liver surgery sequestrate exogenous VEGFA, reduce circulating VEGFA bioavailability, and consequently restrict the opportunity for VEGFA to be taken up by the liver and to exert its protective effects [29, 30]. The concomitant administration of VEGFA with an antibody against sFlt1 ensures that the exogenous VEGFA reaches the steatotic liver in order to protect against damage and regenerative failure.

Herein, we report for the first time that the benefits of VEGFA in non-steatotic livers in the event of PH were maintained when implementing vascular occlusion, which increases our knowledge about the applicability of this growth factor [6, 7, 9]. However, it is important to denote that the effects of VEGFA on the VEGFR2-Id1-Wnt2 pathway in non-steatotic livers subjected to PH were dependent on the presence or absence of vascular occlusion. In fact, in previous studies [7, 43], Id1 was required to induce Wnt2 activation in non-steatotic livers submitted to PH whereas in our hands, Id1 seemed to play a minor role in damage and regeneration in non-steatotic livers under PH+I/R conditions. The pharmacological regulation of VEGFA or its receptor, VEGFR2, affected the degree of damage and regeneration response in non-steatotic livers under PH+I/R conditions independently of changes in Id1 expression. Indeed, VEGFA administration in Ln animals (without the induction of changes in Id1 expression) increased Wnt2 in non-steatotic livers and this was associated with improvements in the levels of damage and regeneration. In addition, antibodies against VEGFR2 abolished the beneficial effects of VEGFA on Wnt2, damage, and the regenerative process. Furthermore, similar to that occurring for VEGFA treatment, the administration of Wnt2 decreased hepatic injury and regeneration failure under PH+I/R conditions, resulting in reduced damage and inflammation in non-steatotic livers and improved liver functionality, regeneration, and survival rate. Taken together, our results demonstrate for the first time that the beneficial effects of VEGFA against damage and regenerative failure in non-steatotic livers under PH+I/R conditions are exerted through the VEGFR2-Wnt2 pathway, independently of Id1.

A previous study indicated that the administration of VEGFA protected diet-induced steatotic livers under PH without I/R [8]. On the other hand, our results indicated that VEGFA administration should not be considered as an effective strategy to reduce damage and improve liver regeneration in surgical conditions requiring both liver regeneration and vascular occlusion. Previous results in obese mice with NAFLD indicated that the down-regulation of Wnt2 signaling was associated with the inhibition of hepatocyte proliferation [44, 45]. However, in our study, Wnt2 seemed to play a minor role in steatotic livers under PH+I/R conditions. Indeed, the concomitant administration of VEGFA and anti-sFlt1 (which increased VEGFA availability in the steatotic liver) did not induce changes in either Id1 or Wnt2, whereas the PI3K/Akt pathway was up-regulated. This increase in PI3K/Akt expression was abolished when we inhibited VEGFA using antibodies against VEGFR2, whereas the levels of both Id1 and Wnt2 were unchanged. Indeed, the inhibition of PI3K/Akt pathway abolished the benefits resulting from VEGFA+anti-sFlt1 treatment on damage and regeneration in steatotic livers. Thus, if sFlt1 action is blocked, VEGFA might exert its effects on steatotic livers by activating the PI3K/Akt rather than the Id1-Wnt2 pathway.

Given our experimental results, circulating sFlt1 may determine VEGFA availability and its effects on target organs. Obviously, intensive investigations (which were not part of the present study) will be necessary to determine whether all these experimental results could be extrapolated to clinical practice in liver surgery or in other pathologies. Yilmaz et al. [46] showed that patients with NAFLD have significantly lower serum sFlt1 concentrations than matched controls. On the other hand, Coulon et al. [47] found that serum levels of sFlt1 were significantly higher in NASH and/or simple steatosis patients compared to controls. The authors indicate that the reason for these different results may lie in the diversity of the patient populations and their clinical characteristics (differences in BMI and inclusion or exclusion of diabetes mellitus patients) or in the differences in laboratory techniques. Indeed, the results reported by Coulon et al. [47] are in line with other papers indicating increases in circulating sFlt1 levels in patients with liver cirrhosis as well as in patients with chronic kidney disease [32, 33, 48].

**Fig. 8:**
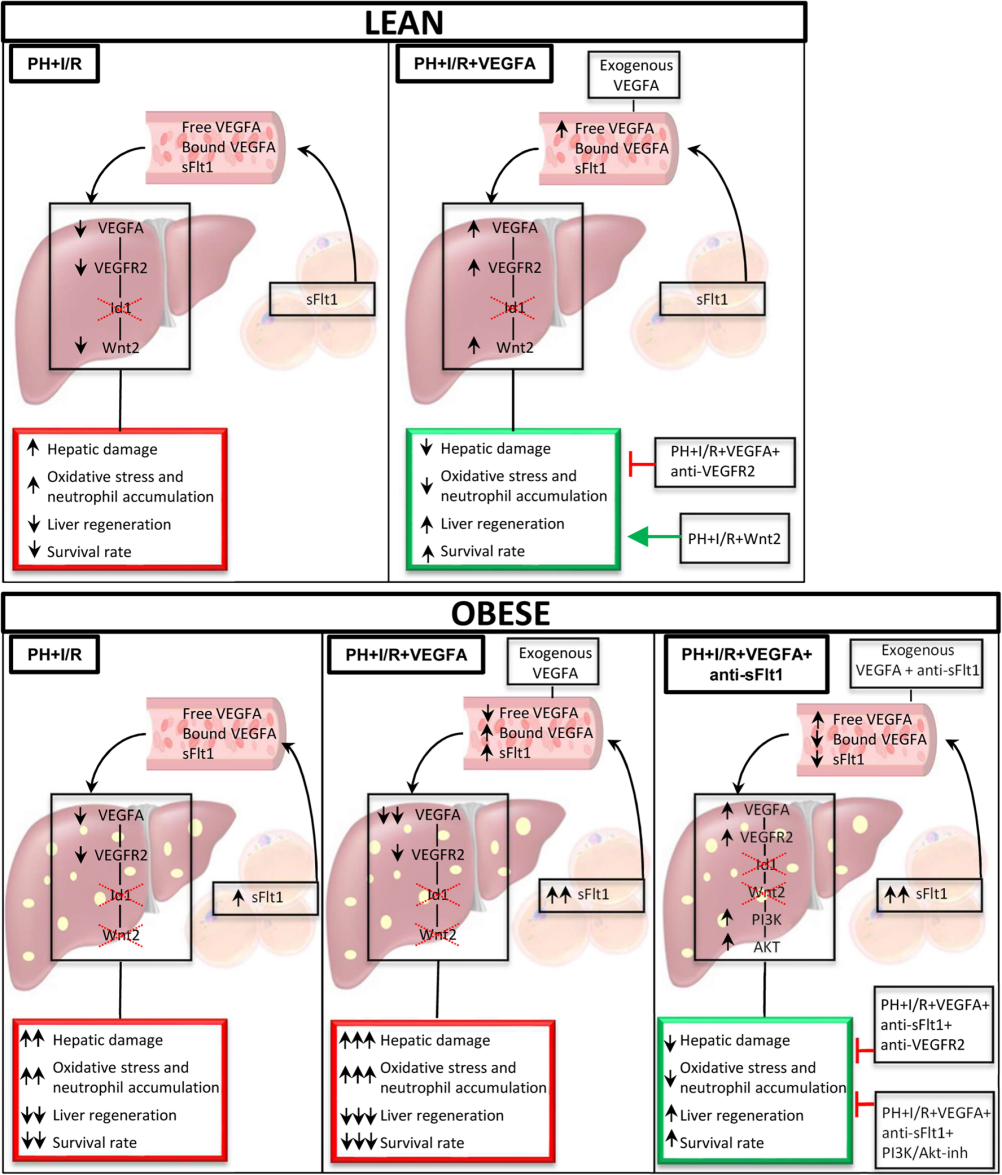
Schematic representation showing the effects of the different interventions, depicting outcomes and proposed signaling pathways of the current study

In conclusion, our experimental results indicate that under PH+I/R conditions, strategies based on treatment with VEGFA alone are probably useful in the attempts to protect non-steatotic livers. However, a pharmacological strategy based on the combination of VEGFA and anti-sFlt1 is required to protect steatotic livers against damage and regenerative failure (Fig. 8). In addition, the present experimental study provides new mechanistic insights into potential therapeutic interventions in the pathology of liver surgery in PH under vascular occlusion, which are also specific for steatotic and non-steatotic livers. These could involve the VEGFA-VEGFR2-Wnt2 pathway for non-steatotic livers and the VEGFA+anti-sFlt1-VEGFR2-PI3K/Akt pathway for steatotic livers.

## Electronic supplementary material

ESM 1 (PDF 1336 kb)

ESM 2 (PDF 584 kb)

ESM 3 (PDF 267 kb)
