# Supplementary material for: Relevance of VEGFA in rat livers subjected to partial hepatectomy under ischemia-reperfusion
Source: J Mol Med (Berl). 2019 Jun 29;97(9):1299–314. doi: 10.1007/s00109-019-01811-y (PMC6713699; doi:10.1007/s00109-019-01811-y)
Supplement: Supplementary file 1 — (PDF 1336 kb) [file 109_2019_1811_MOESM1_ESM.pdf]

ESM_1

RELEVANCE OF VEGFA IN RAT LIVERS SUBJECTED TO PARTIAL HEPATECTOMY UNDER ISCHEMIA-REPERFUSION

JOURNAL OF MOLECULAR MEDICINE

Esther Bujaldon[1]*, María Eugenia Cornide-Petronio[1]*, José Gulfo[2], Floriana Rotondo[1], Cindy Ávalos de León[1], Elsa Negrete-Sánchez[1], Jordi Gracia-Sancho[3], Anna Novials[4,5], Mónica B. Jiménez-Castro[6#], Carmen Peralta[1, 2, 7#]

[1]Institut d'Investigacions Biomèdiques August Pi i Sunyer (IDIBAPS), Barcelona, Spain; [2]Centro de Investigación Biomédica en Red de Enfermedades Hepáticas y Digestivas, Barcelona, Spain. [3]Liver Vascular Biology Research Group, IDIBAPS, CIBEREHD, Barcelona, Spain; [4]Diabetes and Obesity Research Laboratory, Institut d'Investigacions Biomèdiques August Pi i Sunyer (IDIBAPS), Barcelona, Spain; [5]Spanish Biomedical Research Center in Diabetes and Associated Metabolic Disorders (CIBERDEM), Barcelona, Spain; [6]Transplant Biomedicals S.L., Barcelona, Spain; [7]Facultad de Medicina, Universidad International de Cataluña, Barcelona, Spain.

[*] EB and MECP contributed equally to this work (as first author)

[#] MJC and CP contributed equally to this work (as last author)

Correspondence to: Carmen Peralta Uroz

E-mail: cperalta@clinic.ub.es

Telephone: +34932275400 Ext 4177

SUPPLEMENTAL MATERIAL AND METHODS

MATERIAL AND METHODS

Experimental animals

Male homozygous obese (Ob) (400–450 g) and heterozygous lean (Ln) Zucker rats (350–400 g) for the genetic obesity experimental model, and male Sprague Dawley rats (SD) (200–220 g) for the experimental model of steatosis induced by choline-deficient diet (CDD) were purchased from Charles River (Paris, France). Animals were allowed to acclimatize to the animal room conditions for two weeks prior to surgical procedures. Animals were housed with one or two cagemates of the same strain in a standard, shoebox-style polycarbonate cage (24x46x20 cm) with standard stainless-steel lids, hardwood chip bedding and environmental enrichment. The environmental conditions in the animal room were: lighting, 300 to1000 lumens/m2 at cage level and lights on from 0700 to1900; temperature, 20 to 28 ºC); and relative humidity, 30% to 60%. Zucker rats were given water and standard laboratory food (rat chow) ad libitum. Liver steatosis was induced in SD rats by feeding a CDD for 10 days [1, 2]. The diet was purchased from Dyets Inc. (Bethlehem, PA), and its analytical composition (a modified #518753 choline deficient diet, g/kg) was: Alcohol-extracted peanut meal 90, Soy protein isolate (low in choline) 80, L-Cysteine 2, Cellulose fibre 10, Cornstarch 100, Dextrin 100, Sucrose 413, Choline bitartrate 0, Vitamin-free casein 10, Salt mix 35, Primex (hydrogenated vegetable oil) 100, Vitamin mix 10 and Corn oil 50. Control rats (SD) were kept on a standard chow diet containing adequate levels of choline (14.48 g/kg of choline at the expense of sucrose). After 10 days, no differences in rat weight, ranging between 350 and 380 g, were noted in SD and CDD-SD rats.

Ob Zucker and CDD-SD rats showed severe macrovesicular and microvesicular fatty infiltration in hepatocytes (60–70% steatosis) [1-4]. All procedures were started at 0900 and were performed under isofluorane anesthesia. Blood pressure, heart rate, and body temperature were monitored. All procedures were approved by the Laboratory Animal Care and Use Committee of Barcelona University and by the Generalitat de Catalunya. European Union regulations (Directive 86/609 EEC) for animal experiments were respected.

Sample collections

In view of the results of previous studies based on the peaks in the parameters of hepatic injury and liver regeneration [5-8], hepatic injury (transaminases and damage score) and liver regeneration (percentage of Ki67 and cyclins) were determined after 24 h of liver surgery in groups from protocols 1-4. To reinforce

the deleterious effects of VEGFA in obese animals, hepatic damage (plasma AST, ALT, GLDH, ALP and bilirubin levels; and damage score) and liver regeneration (percentage of Ki67 and cyclin E levels) were also determined 72 h after liver surgery in Ob Zucker animals from groups of protocol 1. For survival studies, a sub-group of 20 Ln and 20 Ob Zucker rats were subjected to interventions described for groups corresponding to protocols 1and 3 and the survival was monitored for 14 days [9]. Animals that died in the follow-up period underwent autopsy. The different treatments were administered immediately before surgery and the doses were selected on the basis of previous studies [2, 9-15] and preliminary studies from our group. The interventions and measurements for protocols 1-4 are summarized in Supplementary Figure 1.

Biochemical determinations

Alanine aminotransferase (ALT), aspartate aminotransferase (AST) and glutamate dehydrogenase (GLDH) in plasma and malondialdehyde (MDA) and myeloperoxidase (MPO) in liver were measured as described elsewhere [9, 16, 17]. Alkaline phosphatase (ALP), bilirubin, Von Willebrand factor (vWF), hyaluronic acid (HA) and sFlt1 in plasma were measured using commercial kits (ALP: Abcam, UK; bilirubin: Sigma-Aldrich, USA; vWF and sFlt1: Elabscience Biotechnology, USA; HA: R&D Systems, USA).

Western blotting

Liver and peripheral adipose tissues were homogenized and Western blotting was performed as described elsewhere [10, 18-23], using the antibodies against: VEGFA, p-VEGFR2, Id1, Wnt2, cyclin E, cyclin A, p-AKT (Santa Cruz Biotechnology, USA), PI3K (Cell Signaling Technology, USA), cyclin D1 and β-actin (Sigma-Aldrich, USA). To visualize the bound and the free forms of VEGFA, the same protocol was carried out but with a non-reducing 10% gel [24]. Immunoreactive protein bands were quantified densitometrically with Quantity One software.

RT-PCR

Total RNA was isolated from frozen liver and peripheral adipose tissue using TRIzol Reagent (Invitrogen, USA) and was reverse transcribed using the High Capacity cDNA Reverse Transcription Kits (Applied Biosystems, USA). Real-time PCR was performed with PowerUp SYBR Green Master Mix using primers for: *Vegfa* 5’-CAAACCTCACCAAAGCCAGC-3’ (forward) and 5’-TTCTCCGCTCTGAACAAGGC-3’ (reverse), *Flt1* 5’-GGTGTCTATAGGTGCCGAGC-3’ (forward) and 5’-GGGTGATCAGCTCCAGGTTT-3’ (reverse), s*Flt1* 5’-GAAGACTCGGGCACCTATGC-3’

(forward) and 5’-GCAGTGCTCACCTCTAACGA-3’ (reverse), and *Gapdh* 5’-AGTGCCAGCCTCGTCTCATA-3’ (forward) and 5’-TAACCAGGCGTCCGATACG-3’ (reverse) as control gene. It is well known that sFlt1 is an alternatively spliced, secreted isoform of the cell-surface receptor membrane-bound Flt1. Therefore, sFlt1 lacks the transmembrane and tyrosine kinase domains of Flt1 [24, 25]. In addition, Flt1 can also derive from the endoproteolytic cleavage of Flt1 in endothelial cells [26].

Histology, Red Oil staining, and Immunohistochemistry

Liver and peripheral adipose tissue sections were fixed by immersion into a solution of 10% formaldehyde and paraffin-embedded. To appraise the severity of hepatic injury, we graded hematoxylin and eosin–stained sections with a point counting method on an ordinal scale: (0) minimal or no evidence of injury; (1) mild injury (cytoplasmic vacuolation and focal nuclear pyknosis); (2) moderate to severe injury (extensive nuclear pyknosis, cytoplasmic hypereosinophilia, and a loss of intercellular borders); (3) severe necrosis (disintegration of hepatic cords, haemorrhaging, and neutrophil infiltration); and (4) very severe necrosis (showing the latter manifestations to extreme degree) [27]. Liver steatosis was evaluated via red oil staining [9]. Liver samples were immunostained with a rabbit monoclonal antibody against Ki67 (DAKO, USA). For immunohistochemistry analysis of VEGFA, liver sections were immunostained with a mouse monoclonal antibody against VEGFA (Santa Cruz Biotechnology, USA). For immunohistochemistry analysis of Flt1 and sFlt1, adipose tissue was immunostained with a rabbit monoclonal antibody against sFlt1 and Flt1 (Abcam, UK). Samples were developed with diaminobenzidine, and counterstained with haematoxylin [9]. At least 30 high-power fields per slide were counted. The slides were blinded to the examiners who had extensive experience in evaluating liver pathology. At least four sections were examined per liver and adipose tissue sample.

Statistics

Statistical significance of differing variables was determined via non-parametric Kruskal-Wallis test. Mann-Whitney U test was applied to groups showing significant differences, and adjusted p-values were calculated using False Discovery Rate (FDR) method ($p.adj<0.05$ considered significant). Survival estimates, obtained by Kaplan-Meier method, were then compared using both Log-rank (Mantel-Cox) and Gehan-Breslow-Wilcoxon tests, since Gehan-Breslow-Wilcoxon test emphasizes early differences [28], with statistical significance set at $p<0.05$.

REFERENCES

[1] Vendemiale G, Grattagliano I, Caraceni P, Caraccio G, Domenicali M, Dall'Agata M, Trevisani F, Guerrieri F, Bernardi M, Altomare E (2001) Mitochondrial oxidative injury and energy metabolism alteration in rat fatty liver: effect of the nutritional status. Hepatology 33:808-815. https://doi.org/10.1053/jhep.2001.23060

[2] Mendes-Braz M, Elias-Miró M, Kleuser B, Fayyaz S, Jiménez-Castro MB, Massip-Salcedo M, Gracia-Sancho J, Ramalho FS, Rodes J, Peralta C (2014) The effects of glucose and lipids in steatotic and non-steatotic livers in conditions of partial hepatectomy under ischaemia-reperfusion. Liver Int 34:e271-289. https://doi.org/10.1111/liv.12348

[3] Caraceni P, Nardo B, Domenicali M, Turi P, Vici M, Simoncini M, De Maria N, Trevisani F, Van Thiel DH, Derenzini M, Cavallari A, Bernardi M (1999) Ischemia-reperfusion injury in rat fatty liver: role of nutritional status. Hepatology 29:1139-1146. https://doi.org/10.1002/hep.510290407

[4] Hakamada K, Sasaki M, Takahashi K, Umehara Y, Konn M (1997) Sinusoidal flow block after warm ischemia in rats with diet-induced fatty liver. J Surg Res 70:12-20. https://doi.org/10.1006/jsre.1997.5077

[5] Rozga J (2002) Hepatocyte proliferation in health and in liver failure. Med Sci Monit 8:RA32-38.

[6] Maruyama H, Harada A, Kurokawa T, Kobayashi H, Nonami T, Nakao A, Takagi H 1995) Duration of liver ischemia and hepatic regeneration after hepatectomy in rats. J Surg Res 58:290-294. https://doi.org/10.1006/jsre.1995.1045

[7] Asakawa H, Jeppsson B, Mack P, Hultberg B, Hägerstrand I, Bengmark S (1989) Acute ischemic liver failure in the rat: a reproducible model not requiring portal decompression. Eur Surg Res 21:42-48. https://doi.org/10.1159/000129002

[8] Ferris GM, Clark JB. Nicotinamide nucleotide synthesis in regenerating rat liver (1971) Biochem J 121:655-662.

[9] Cornide-Petronio ME, Bujaldon E, Mendes-Braz M, Avalos de León CG, Jiménez-Castro MB, Álvarez-Mercado AI, Gracia-Sancho J, Rodés J, Peralta C (2017) The impact of cortisol in steatotic and non-steatotic liver surgery. J Cell Mol Med 21:2344-2358. https://doi.org/10.1111/jcmm.13156

[10] McCormack L, Petrowsky H, Jochum W, Furrer K, Clavien PA (2007) Hepatic steatosis is a risk factor for postoperative complications after major hepatectomy: a matched case-control study. Ann Surg 245:923-930. https://doi.org/10.1097/01.sla.0000251747.80025.b7

[11] Cornide-Petronio ME, Bujaldon E, Mendes-Braz M, Avalos de León CG, Jiménez-Castro MB, Álvarez-Mercado AI, Gracia-Sancho J, Rodés J, Peralta C (2017) The impact of cortisol in steatotic and non-steatotic liver surgery. J Cell Mol Med 21:2344-2358. https://doi.org/10.1111/jcmm.13156

[12] Bockhorn M, Goralski M, Prokofiev D, Dammann P, Grünewald P, Trippler M, Biglarnia A, Kamler M, Niehues EM, Frilling A, Broelsch CE, Schlaak JF (2007) VEGF is important for early liver regeneration after partial hepatectomy. J Surg Res 138:291-299. https://doi.org/10.1016/j.jss.2006.07.027

[13] Kommareddy S, Amiji M (2007) Antiangiogenic gene therapy with systemically administered sFlt-1 plasmid DNA in engineered gelatin-based nanovectors. Cancer Gene Ther 14:488-498. https://doi.org/10.1038/sj.cgt.7701041

[14] Wedge SR, Ogilvie DJ, Dukes M, Kendrew J, Chester R, Jackson JA, Boffey SJ, Valentine PJ, Curwen JO, Musgrove HL, Graham GA, Hughes GD, Thomas AP, Stokes ES, Curry B, Richmond GH, Wadsworth PF, Bigley AL, Hennequin LF (2002) ZD6474 inhibits vascular endothelial growth factor signaling, angiogenesis, and tumor growth following oral administration. Cancer Res 62:4645-4655.

[15] Launay JM, Mouillet-Richard S, Baudry A, Pietri M, Kellermann O (2011) Raphe-mediated signals control the hippocampal response to SRI antidepressants via miR-16. Transl Psychiatry 1:e56. https://doi.org/10.1038/tp.2011.54

[16] O'Brien PJ, Slaughter MR, Polley SR, Kramer K (2002) Advantages of glutamate dehydrogenase as a blood biomarker of acute hepatic injury in rats. Lab Anim 36:313-321. https://doi.org/10.1258/002367702320162414

[17] Jiménez-Castro MB, Meroño N, Mendes-Braz M, Gracia-Sancho J, Martínez-Carreres L, Cornide-Petronio ME, Casillas-Ramirez A, Rodés J, Peralta C (2015) The effect of brain death in rat steatotic and non-steatotic liver transplantation with previous ischemic preconditioning. J Hepatol 62:83-91. https://doi.org/10.1016/j.jhep.2014.07.031

[18] Tsuchihashi S, Ke B, Kaldas F, Flynn E, Busuttil RW, Briscoe DM, Kupiec-Weglinski JW (2006) Vascular endothelial growth factor antagonist modulates leukocyte trafficking and protects mouse livers against ischemia/reperfusion injury. Am J Pathol 168:695-705. https://doi.org/10.2353/ajpath.2006.050759

[19] Albanese C, Johnson J, Watanabe G, Eklund N, Vu D, Arnold A, Pestell RG (1995) Transforming p21ras mutants and c-Ets-2 activate the cyclin D1 promoter through distinguishable regions. J Biol Chem 270:23589-23597.

[20] Wiercinska E, Wickert L, Denecke B, Said HM, Hamzavi J, Gressner AM, Thorikay M, ten Dijke P, Mertens PR, Breitkopf K, Dooley S (2006) Id1 is a critical mediator in TGF-beta-induced transdifferentiation of rat hepatic stellate cells. Hepatology 43:1032-1041. https://doi.org/10.1002/hep.21135

[21] Maharaj AS, Saint-Geniez M, Maldonado AE, D'Amore PA (2006) Vascular endotelial growth factor localization in the adult. Am J Pathol 168:639-648. https://doi.org/10.2353/ajpath.2006.050834

[22] Klein D, Demory A, Peyre F, Kroll J, Augustin HG, Helfrich W, Kzhyshkowska J, Schledzewski K, Arnold B, Goerdt S (2008) Wnt2 acts as a cell type-specific, autocrine growth factor in rat hepatic sinusoidal endothelial cells cross-stimulating the VEGF pathway. Hepatology 47:1018-1031. https://doi.org/10.1002/hep.22084

[23] Lee YJ, Karl DL, Maduekwe UN, Rothrock C, Ryeom S, D'Amore PA, Yoon SS (2010) Differential effects of VEGFR-1 and VEGFR-2 inhibition on tumor metastases based on host organ environment. Cancer Res 70:8357-8367. https://doi.org/10.1158/0008-5472.CAN-10-1138

[24] Ambati BK, Nozaki M, Singh N, Takeda A, Jani PD, Suthar T, Albuquerque RJ, Richter E, Sakurai E, Newcomb MT, Kleinman ME, Caldwell RB, Lin Q, Ogura Y, Orecchia A, Samuelson DA, Agnew DW, St Leger J, Green WR, Mahasreshti PJ, Curiel DT, Kwan D, Marsh H, Ikeda S, Leiper LJ, Collinson JM, Bogdanovich S, Khurana TS, Shibuya M, Baldwin ME, Ferrara N, Gerber HP, De Falco S, Witta J, Baffi JZ, Raisler BJ, Ambati J (2006) Corneal avascularity is due to soluble VEGF receptor-1. Nature 443:993-997. https://doi.org/10.1038/nature05249

[25] Kendall RL, Thomas KA (1993) Inhibition of vascular endothelial cell growth factor activity by an endogenously encoded soluble receptor. Proc Natl Acad Sci USA 90:10705-10709.

[26] Mezquita B, Mezquita P, Pau M, Mezquita J, Mezquita C (2014) Unlocking Doors without Keys: Activation of Src by Truncated C-terminal Intracellular Receptor Tyrosine Kinases Lacking Tyrosine Kinase Activity. Cells 3:92-111. https://doi.org/10.3390/cells3010092.

[27] Camargo CA Jr, Madden JF, Gao W, Selvan RS, Clavien PA (1997) Interleukin-6 protects liver against warm ischemia/reperfusion injury and promotes hepatocyte proliferation in the rodent. Hepatology 26:1513-1520.

[28] Guerrero M, Crisafulli E, Liapikou A, Huerta A, Gabarrús A, Chetta A, Soler N, Torres A (2016) Readmission for Acute Exacerbation within 30 Days of Discharge Is Associated with a Subsequent Progressive Increase in Mortality Risk in COPD Patients: A Long-Term Observational Study. PLoS One 11:e0150737. https://doi.org/10.1371/journal.pone.0150737.

SUPPLEMENTARY FIGURE LEGENDS

Supplementary Figure 1. Flow chart of the interventions and measurements corresponding to protocols 1-4. Protocol 1) VEGFA impact and availability in Ln and Ob Zucker rats undergoing PH+I/R; Protocol 2) Role of adipose tissue in the circulating sFlt1 levels in Ln and Ob Zucker rats undergoing PH+I/R; Protocol 3) Underlying mechanisms of VEGFA in Ln and Ob Zucker rats undergoing PH+I/R; Protocol 4) VEGFA impact and availability in SD and CDD-SD rats undergoing PH+I/R.

Experimental desing

Protocol 1.

1. **Sham** (Ln and Ob Zucker rats) — Hepatic hiliar vessels dissected
2. **PH+I/R** (Ln and Ob Zucker rats) — Partial hepatectomy + Ischemia-Reperfusion
3. **PH+I/R+VEGFA** (Ln and Ob Zucker rats) — Partial hepatectomy + Ischemia-Reperfusion; ↑ *VEGFA*
4. **PH+I/R+VEGFA+anti-sFlt1** (Ob Zucker rats) — Partial hepatectomy + Ischemia-Reperfusion; ↑ *VEGFA+anti-sFlt1*

Protocol 2.

5. **Sham+LPT** (Ln and Ob Zucker rats) — Lipectomy; Hepatic hiliar vessels dissected
6. **PH+I/R+LPT** (Ln and Ob Zucker rats) — Lipectomy; Partial hepatectomy + Ischemia-Reperfusion
7. **PH+I/R+VEGFA+LPT** (Ln and Ob Zucker rats) — ↑ *VEGFA*; Lipectomy; Partial hepatectomy + Ischemia-Reperfusion

Protocol 3.

8. **PH+I/R+VEGFA+anti-VEGFR2** (Ln Zucker rats) — Partial hepatectomy + Ischemia-Reperfusion; ↑ *VEGFA+anti-VEGFR2*
9. **PH+I/R+Wnt2** (Ln Zucker rats) — Partial hepatectomy + Ischemia-Reperfusion; ↑ *Wnt2*
10. **PH+I/R+VEGFA+anti-sFlt1+anti-VEGFR2** (Ob Zucker rats) — Partial hepatectomy + Ischemia-Reperfusion; ↑ *VEGFA+anti-sFlt1+anti-VEGFR2*
11. **PH+I/R+VEGFA+anti-sFlt1+PI3K/Akt-inh** (Ob Zucker rats) — Partial hepatectomy + Ischemia-Reperfusion; ↑ *VEGFA+anti-sFlt1+PI3K/Akt-inh*

Protocol 4.

12. **Sham** (SD and CDD-SD rats) — Hepatic hiliar vessels dissected
13. **PH+I/R** (SD and CDD-SD rats) — Partial hepatectomy + Ischemia-Reperfusion
14. **PH+I/R+VEGFA** (SD and CDD-SD rats) — Partial hepatectomy + Ischemia-Reperfusion; ↑ *VEGFA*
15. **PH+I/R+VEGFA+anti-sFlt1** (CDD-SD rats) — Partial hepatectomy + Ischemia-Reperfusion; ↑ *VEGFA+anti-sFlt1*

Test at 24 h and 72 h after liver surgery. Plasmatic VEGFA, transaminases, GLDH, ALP, Bilirubin, vWF, HA, sFlt1. Hepatic VEGFA, damage score, MDA, MPO, Ki-67 positive hepatocytes, cyclines, sFlt1, Flt1, VEGFR2, Id1, Wnt2, PI3K, Akt. sFlt1, Flt1, VEGFA in adipose tissue. Survival was monitored for 14 days.
