# Supplementary material for: Relevance of VEGFA in rat livers subjected to partial hepatectomy under ischemia-reperfusion
Source: J Mol Med (Berl). 2019 Jun 29;97(9):1299–314. doi: 10.1007/s00109-019-01811-y (PMC6713699; doi:10.1007/s00109-019-01811-y)
Supplement: Supplementary file 2 — (PDF 584 kb) [file 109_2019_1811_MOESM2_ESM.pdf]

**ESM_2**

# RELEVANCE OF VEGFA IN RAT LIVERS SUBJECTED TO PARTIAL HEPATECTOMY UNDER ISCHEMIA-REPERFUSION

**JOURNAL OF MOLECULAR MEDICINE**

Esther Bujaldon[1]*, María Eugenia Cornide-Petronio[1]*, José Gulfo[2], Floriana Rotondo[1], Cindy Ávalos de León[1], Elsa Negrete-Sánchez[1], Jordi Gracia-Sancho[3], Anna Novials[4,5], Mónica B. Jiménez-Castro[6#], Carmen Peralta[1, 2, 7#]

[1]Institut d'Investigacions Biomèdiques August Pi i Sunyer (IDIBAPS), Barcelona, Spain; [2]Centro de Investigación Biomédica en Red de Enfermedades Hepáticas y Digestivas, Barcelona, Spain. [3]Liver Vascular Biology Research Group, IDIBAPS, CIBEREHD, Barcelona, Spain; [4]Diabetes and Obesity Research Laboratory, Institut d'Investigacions Biomèdiques August Pi i Sunyer (IDIBAPS), Barcelona, Spain; [5]Spanish Biomedical Research Center in Diabetes and Associated Metabolic Disorders (CIBERDEM), Barcelona, Spain; [6]Transplant Biomedicals S.L., Barcelona, Spain; [7]Facultad de Medicina, Universidad International de Cataluña, Barcelona, Spain.

[*] EB and MECP contributed equally to this work (as first author)

[#] MJC and CP contributed equally to this work (as last author)

Correspondence to: Carmen Peralta Uroz

E-mail: cperalta@clinic.ub.es

Telephone: +34932275400 Ext 4177

**SUPPLEMENTARY FIGURE LEGENDS**

**Supplementary Figure 2.** Effect of VEGFA on hepatic damage and regeneration in Ob Zucker rats undergoing PH+I/R 72 h after surgery. (A) Plasma AST, ALT, GLDH, damage score, ALP and bilirubin levels. (B) Hepatic regeneration (percentage of Ki67-positive-hepatocytes, representative photomicrographs of Ki-67 immunohistochemical positivity (10x) and cyclin E levels). +P < 0.05 versus PH+I/R.

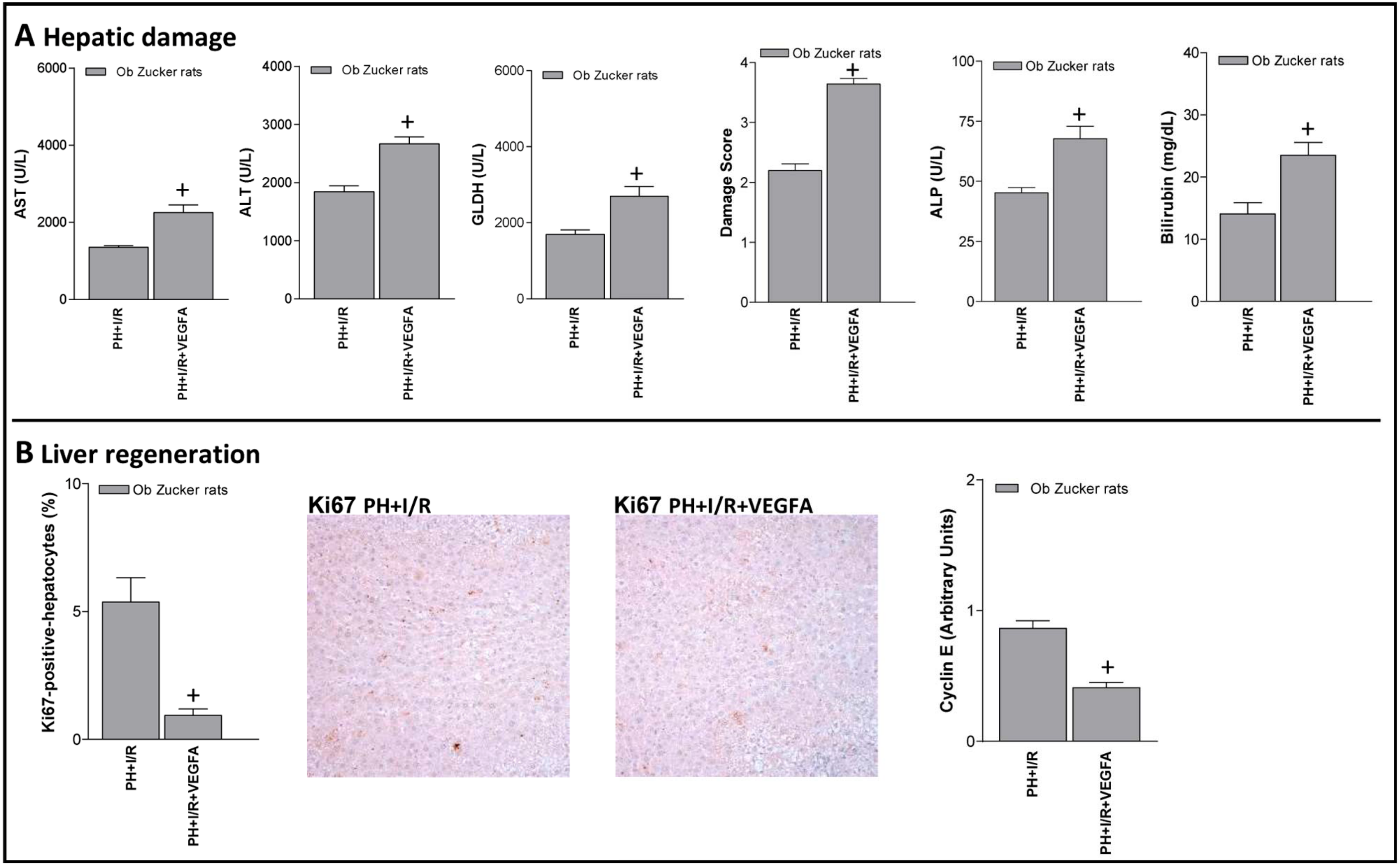
A Hepatic damage
Ob Zucker rats
AST (U/L)
ALT (U/L)
GLDH (U/L)
Damage Score
ALP (U/L)
Bilirubin (mg/dL)
PH+I/R
PH+I/R+VEGFA
+
B Liver regeneration
Ki67-positive-hepatocytes (%)
Ki67 PH+I/R
Ki67 PH+I/R+VEGFA
Cyclin E (Arbitrary Units)
