# Supplementary material for: Relevance of VEGFA in rat livers subjected to partial hepatectomy under ischemia-reperfusion
Source: J Mol Med (Berl). 2019 Jun 29;97(9):1299–314. doi: 10.1007/s00109-019-01811-y (PMC6713699; doi:10.1007/s00109-019-01811-y)
Supplement: Supplementary file 3 — (PDF 267 kb) [file 109_2019_1811_MOESM3_ESM.pdf]

ESM_3

RELEVANCE OF VEGFA IN RAT LIVERS SUBJECTED TO PARTIAL HEPATECTOMY UNDER ISCHEMIA-REPERFUSION

JOURNAL OF MOLECULAR MEDICINE

Esther Bujaldon[1]*, María Eugenia Cornide-Petronio[1]*, José Gulfo[2], Floriana Rotondo[1], Cindy Ávalos de León[1], Elsa Negrete-Sánchez[1], Jordi Gracia-Sancho[3], Anna Novials[4,5], Mónica B. Jiménez-Castro[6#], Carmen Peralta[1, 2, 7#]

[1]Institut d'Investigacions Biomèdiques August Pi i Sunyer (IDIBAPS), Barcelona, Spain; [2]Centro de Investigación Biomédica en Red de Enfermedades Hepáticas y Digestivas, Barcelona, Spain. [3]Liver Vascular Biology Research Group, IDIBAPS, CIBEREHD, Barcelona, Spain; [4]Diabetes and Obesity Research Laboratory, Institut d'Investigacions Biomèdiques August Pi i Sunyer (IDIBAPS), Barcelona, Spain; [5]Spanish Biomedical Research Center in Diabetes and Associated Metabolic Disorders (CIBERDEM), Barcelona, Spain; [6]Transplant Biomedicals S.L., Barcelona, Spain; [7]Facultad de Medicina, Universidad International de Cataluña, Barcelona, Spain.

[*] EB and MECP contributed equally to this work (as first author)

[#] MJC and CP contributed equally to this work (as last author)

Correspondence to: Carmen Peralta Uroz

E-mail: cperalta@clinic.ub.es

Telephone: +34932275400 Ext 4177

SUPPLEMENTARY FIGURE LEGEND

Supplementary Figure 3. Impact of VEGFA on hepatic damage and liver regeneration 24 h after surgery and survival rate in SD and CDD-SD rats. (A) Protein levels of hepatic VEGFA (Representative Western blots at the top and densitometric analysis at the bottom) and sFlt1 levels in plasma and adipose tissue. (B) Hepatic injury (plasma AST, ALT and GLDH levels and damage score). (C) Hepatic regeneration (percentage of Ki67-positive-hepatocytes and cyclins D1, E and A levels). *P < 0.05 versus sham; +P < 0.05 versus PH+I/R.

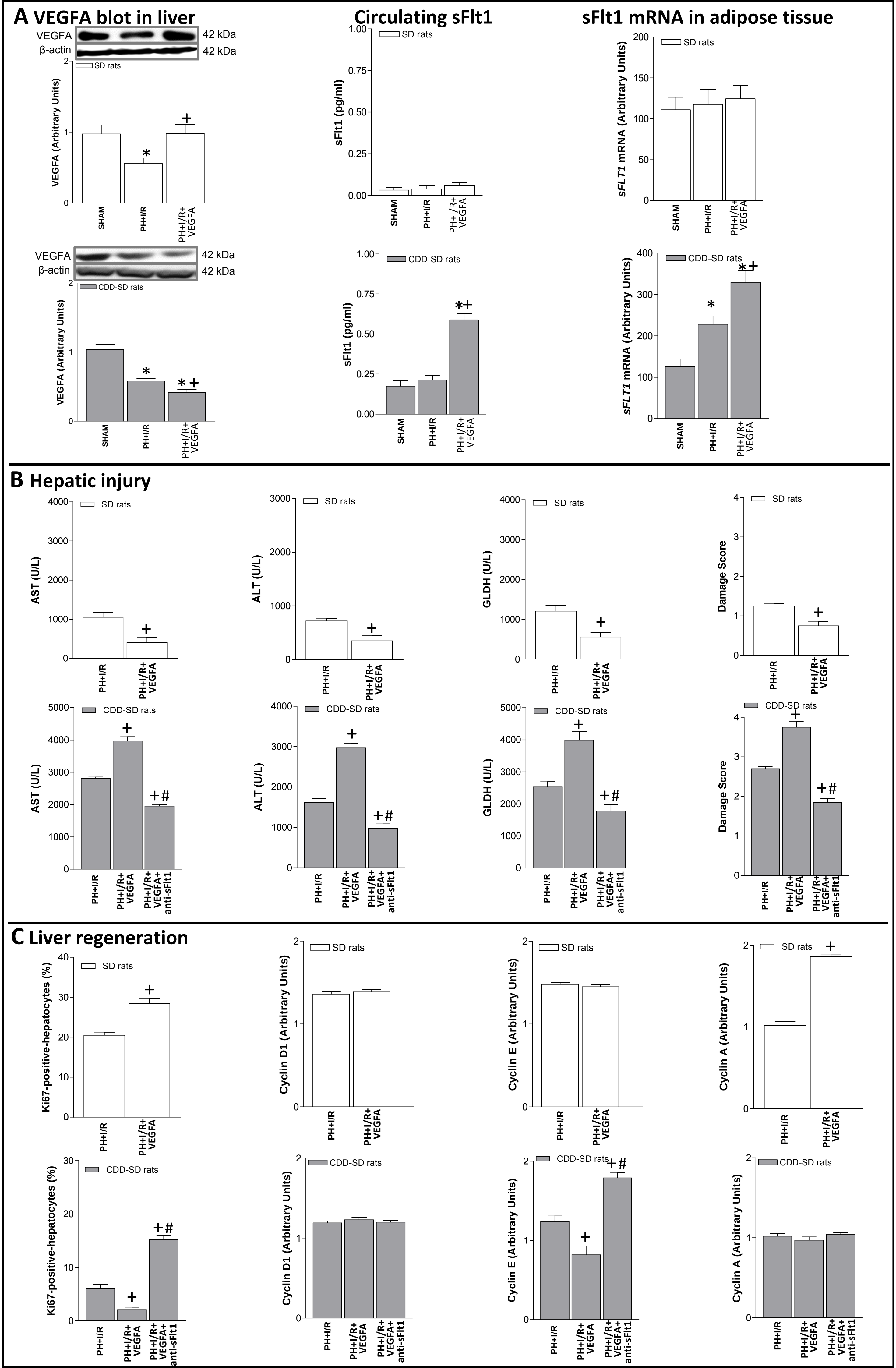
A VEGFA blot in liver
VEGFA
β-actin
42 kDa
42 kDa
SD rats
VEGFA (Arbitrary Units)
SHAM
PH+I/R
PH+I/R+ VEGFA
CDD-SD rats
Circulating sFlt1
sFlt1 (pg/ml)
sFlt1 mRNA in adipose tissue
sFLT1 mRNA (Arbitrary Units)
B Hepatic injury
AST (U/L)
ALT (U/L)
GLDH (U/L)
Damage Score
PH+I/R+ VEGFA+ anti-sFlt1
C Liver regeneration
Ki67-positive-hepatocytes (%)
Cyclin D1 (Arbitrary Units)
Cyclin E (Arbitrary Units)
Cyclin A (Arbitrary Units)
